# Antimicrobial Susceptibility and Genomic Structure of *Arcobacter skirrowii* Isolates

**DOI:** 10.3389/fmicb.2018.03067

**Published:** 2018-12-14

**Authors:** Ingrid Hänel, Helmut Hotzel, Herbert Tomaso, Anne Busch

**Affiliations:** Institute of Bacterial Infections and Zoonoses (IBIZ), Friedrich Loeffler Institute, Jena, Germany

**Keywords:** *Arcobacter skirrowii*, *Arcobacter butzleri*, antimicrobial susceptibility, genomic structure, antibiotic, genome, antibiotic susceptibility, animal health

## Abstract

*Campylobacter* spp. are considered the most common bacterial cause of foodborne gastroenteritis in the world. The family *Campylobacteraceae* includes the genus *Arcobacter* with the three species *Arcobacter butzleri*, *Arcobacter cryaerophilus*, and *Arcobacter skirrowii* as emergent enteropathogens and potential zoonotic agents. Here, we characterized genome sequences of *Arcobacter* that were isolated from water poultry on farms in Germany. Isolates were cultured, identified by MALDI-TOF MS and identification was verified with PCR assays. Antibiotic susceptibility testing of isolates was carried out with erythromycin, ciprofloxacin, doxycycline, tetracycline, gentamicin, and streptomycin using the gradient strip method (*E*-test). We also sequenced whole genomes and predicted antibiotic resistance determinants, virulence factors, performed a phylogenetic analysis to determine the genetic relatedness of these isolates and searched for plasmids.

## Introduction

*Campylobacter* spp. are considered to be the most common bacterial cause of human gastroenteritis in the world (WHO, 2018). In 1991, the new genus *Arcobacter* *(A.)* was introduced within the family of *Campylobacteraceae*. Three species, *Arcobacter butzleri*, *Arcobacter cryaerophilus*, and *Arcobacter skirrowii*, are considered potential zoonotic agents (Prouzet-Mauleon et al., 2006; Levican et al., 2013; Van den Abeele et al., 2014, 2016). *Arcobacter* spp. are present in the digestive tract of healthy animals (Ünver et al., 2013), but are also associated with enteritis and reproductive disorders in animals (De Smet et al., 2012). *Arcobacter* can be transmitted to humans by contaminated food (e.g., poultry products) and water (Collado and Figueras, 2011; Hänel et al., 2016).

Isolation of *A. skirrowii* is often difficult due to its special growth requirements and data on the antibiotic susceptibility of the bacteria are scarce (Yesilmen et al., 2014). Only few representative genomes (for example NCBI, BioProject: PRJNA307998, BioSample: SAMN04386098) have been described.

The aim of this study was to determine the antimicrobial susceptibility of *A. skirrowii* isolated from domestic water poultry to six antibiotics commonly used to treat diarrhea in humans. The genomic features of *A. skirrowii* isolates were analyzed to improve diagnostic and antibiotic treatment options.

## Materials and Methods

*Arcobacter* isolates were cultivated from fecal samples collected in water poultry farms in Thuringia, Germany. A two-step enrichment procedure was done in *Arcobacter* broth (Oxoid, Wesel, Germany) supplemented with antibiotics (cefoperazone, amphotericin and teicoplanin; CAT, Oxoid) under microaerobic conditions (5% O_2_, 10% CO_2_, and 85% N_2_) for 48 h at 30°C. Subsequently, the broth was streaked on plates (Mueller-Hinton/CAT/5% defibrinated bovine blood) and incubated under microaerobic conditions for another 24–48 h at 30°C. Suspicious colonies were recultivated and identified by matrix-assisted laser desorption/ionization time-of-flight mass spectrometry (MALDI-TOF MS) as described before (El-Ashker et al., 2015; Busch et al., 2018). IVD Bacterial Test Standard, Biotyper 3.1 software, and the database DB 4613 (all Bruker Daltonik GmbH, Bremen, Germany) containing spectra of all *Arcobacter* species were used. A confirmation of the species identification was performed using a multiplex PCR assay (Douidah et al., 2014).

Antimicrobial susceptibility to six antibiotics (erythromycin, ciprofloxacin, doxycycline, tetracycline, gentamicin, and streptomycin) was determined using the gradient strip diffusion method (*E*-test^TM^, bioMérieux, Nürtingen, Germany) following the manufacturer’s instructions (Table 1). The bacterial suspensions for the *E*-test were adjusted to an optical density of 0.1 at 600 nm (corresponding to approximately 3 to 5 × 10^8^ cfu/ml) in PBS. 750 μl were evenly spread on a MH agar plate and a single strip was put on each plate. After 48 h of incubation at 30°C under microaerobic conditions, the minimum inhibitory concentration (MIC) was determined. The type strain of *A. skirrowii* DSM 7302 was used as control. For erythromycin, ciprofloxacin, doxycycline, and tetracycline interpretative criteria were based upon EUCAST breakpoints for *Campylobacter*. For gentamicin EUCAST *Enterobacteriaceae* breakpoints were applied. For streptomycin the cut-off value for *Campylobacter jejuni* was used as suggested by the EFSA–Working Group (European Food Safety Authority [EFSA], 2008). The phenotypes were classified as sensitive (S), resistant (R), or intermediate (I) (Table 1).

**Table 1:** Antibiotic susceptibility of *Arcobacter* spp.

	17-1201-3		17-1201-4		17-1206-2		17-1208-1		17-1208-2		17-1168	
	*A. skirrowii*		*A. skirrowii*		*A. skirrowii*		*A. skirrowii*		*A. skirrowii*		*A. butzleri*	
	1	2	1	2	1	2	1	2	1	2	1	2
Erythromycin	*S*	No	*S*	No	*S*	No	*S*	No	*S*	No	*R*	No
*S* ≤ 4mg/L	0.25		1.0		0.25		1.0		0.5		8.0	
*R* > 4 mg/L												
Ciprofloxacin	*S*	No	*S*	No	*S*	No	*S*	No	*S*	No	*S*	No
*S* ≤ 0,5 mg/L	0.03		0.25		0.06		0.25		0.06		0.5	
*R* > 0,5 mg/L												
Doxycyclin	*S*	No	*S*	No	*S*	No	*S*	No	*S*	No	*S*	No
*S* ≤ 2 mg/L	0.064		0.25		0.125		0.50		0.19		2.0	
*R* > 2 mg/L												
Tetracyclin	*S*	No	*S*	No	*S*	No	*S*	No	*S*	No	*R*	No
*S* ≤ 2mg/L	0.12		1.0		0.25		1.0		0.25		4.0	
*R* > 2 mg/L												
Gentamicin	*S*	No	*S*	No	*S*	No	*S*	No	*S*	No	*I*	No
*S* ≤ 2 mg/L	0.5		2.0		1.0		2.0		1.0		4.0	
*I* = 4 mg/L												
*R* > 4 mg/L												
Streptomycin	*R*	No	*R*	No	*R*	No	*R*	No	*R*	No	*R*	No
*S* ≤ 2 mg/L	3.0		8.0		3.0		8.0		4.0		12.0	
*R* > 2 mg/L												


*EUCAST breakpoints for Campylobacter jejuni were used for erythromycin, ciprofloxacin, doxycycline, tetracycline, and gentamicin. EFSA breakpoints were applied for streptomycin (European Food Safety Authority [EFSA], 2008). S, susceptible; R, resistant; I, intermediate; 1, laboratory results; 2, ResFinder prediction.*

**FIGURE 1:**
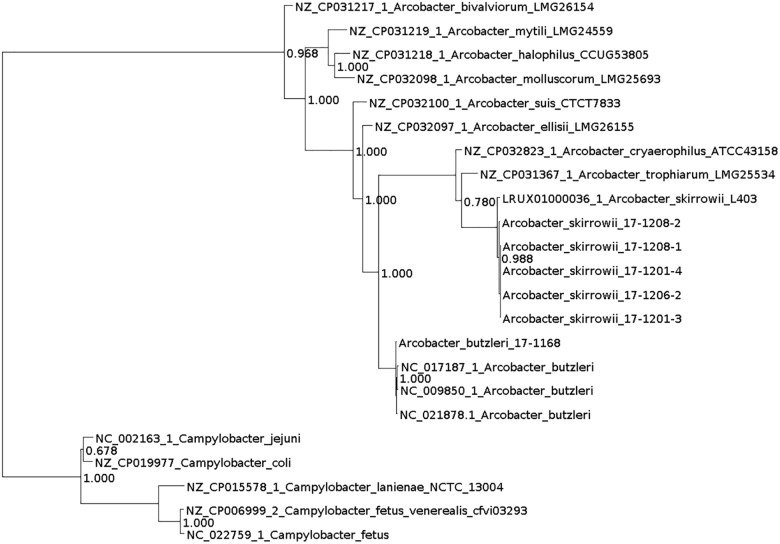
Phylogenetic tree generated with PhyloPhlAn and visualized with Dendroscope.

DNA for whole genome sequencing (WGS) was prepared from colonies harvested from plates. DNA was purified (High Pure PCR Template Preparation Kit; Roche Diagnostics, Mannheim, Germany) and sequencing libraries were generated using the Nextera XT DNA Library Prep Kit (Illumina, Inc., San Diego, CA, United States). From an Illumina MiSeq run 111,000–8,300,000 paired-end reads were generated (mean sequencing depth: 29–216 reads). The assignment of the taxonomic labels to all reads was performed with MetaPhlAn (Segata et al., 2012) and Kraken version 0.10.6 (Wood and Salzberg, 2014). Further read processing included quality trimming and assembly with SPAdes 3.9.1 (–careful) (Bankevich et al., 2012) and filtering by removing contigs with a coverage < 5 and a length < 500. Quality was assessed with QUAST 4.3 (Gurevich et al., 2013). Annotation was performed with Prokka using the recommended standard settings (Seemann, 2014). Additionally, PhyloPhlAn was used to assign microbial phylogeny (Segata et al., 2013) and visualized with Dendroscope (Huson et al., 2007; Figure 1). Subsystem category distribution of the assemblies was done with RAST and SEED (Supplementary Figure 1; Aziz et al., 2008; Brettin et al., 2015). Based on ARIBA in the standard settings (Hunt et al., 2017) several databases were used to identify single nucleotide polymorphisms directly from short reads. Resistance genes were predicted using the ResFinder (Zankari et al., 2012) and the PlasmidFinder was used for the analysis of plasmids (Carattoli et al., 2014). Multilocus Sequence Typing (MLST) was done using the MLST database (Carattoli et al., 2014) and for detection of virulence factors VFDB_full was used (Chen et al., 2005). No reads mapped to reference genes. Therefore, no local assemblies were run. Virulence-associated genes known from the literature, i.e., the genes *ciaB* (HF935951)*, cj1349 (*HF935963), and *cadF* (HF935942) were mapped with Geneious (Kearse et al., 2012) on all assemblies and to the known *A. skirrowii* sequence LRUX01000036.1. Further search for plasmids was done with Bandage (Wick et al., 2015).

All isolates were submitted to the Leibniz Institute, DSMZ-German Collection of Microorganisms and Cell Cultures^1^, and are available under the following DSM numbers: DSM 107942 (*A. butzleri* FLI 17-1168), DSM 107960 (*A. skirrowii* FLI 17-1208-1), DSM 107961 (*A. skirrowii* FLI 17-1201-3), DSM 107962 (*A. skirrowii* FLI 17-1208-2), DSM 107963 (*A. skirrowii* FLI 17-1201-4), DSM 107964 (*A. skirrowii* FLI 17-1206-2). Whole-genome sequences, assemblies, and raw data of all isolates were submitted under the BioProject PRJNA464281.

## Results

Five *A. skirrowii* and one *A. butzleri* isolates were cultivated and identified by MALDI-TOF MS, and due to scores <2,3 confirmed by a multiplex PCR assay. As an example, the MALDI-TOF MS mass list of a wildtyp *A. skirrowii* 1208_2 is given in Supplementary Table 4. Reference spectra were generated for *A. skirrowii* wild type isolates (spectra are available upon request).

Taxonomic analysis of the WGS data with MetaPhlAn and Kraken resulted mostly in “unclassified *Arcobacter”* for *A. skirrowii* isolates, whereas *A. butzleri* could be assigned to the correct species. Also analyses based on the genomic features by sequence identity with RAST showed only a relationship to *A. butzleri* RM4018 for all isolates. A correct taxonomic differentiation of the species was possible with PhyloPhlAn.

The antibiotic susceptibility test results for the *Arcobacter* isolates obtained by *E*-test are shown in Table 1. All *A. skirrowii* isolates were susceptible to erythromycin, ciprofloxacin, doxycycline, tetracycline, and gentamicin and showed resistance to streptomycin. The *A. butzleri* isolate was susceptible to ciprofloxacin and doxycycline, showed intermediate resistance to gentamicin and was resistant to erythromycin, tetracycline, and streptomycin. Based on the WGS data no resistance determinants were predicted with the ResFinder.

The mean coverage obtained for the isolates using WGS was >170 except for *A. skirrowii* isolate 17-1201-3 with a mean coverage of only 20. All sequences were assembled and annotated (Supplementary Tables 1, 2). Assemblies consisted of 36 to 145 contigs. Sequence length was predicted to be between 1,911,841 and 1,940,887 concordant bases. Due to the lower coverage of *A. skirrowii* isolate 17-1201-3 the assembly resulted in more contigs, but genome sizes of all *A. skirrowii* were estimated to be around 1.9 million bases with approximately 1,980 coding sequences. The *A. butzleri* isolate 17-1168 was estimated to have a genome size of 2.2 million bases and included more coding sequences (2,143) than the *A. skirrowii* isolates. The GC content was determined to be 27.6% for *A. skirrowii* and 26.9% for *A. butzleri*, respectively. When the programs were applied with standard settings, no reads mapped to reference genes in the database, therefore no local assemblies were run. However, the known virulence-associated genes (*ciaB* (HF935951)*, cj1349 (*HF935963), *hecA* (HF935064), and *cadF* (HF935942)) could be mapped to all assemblies and the published *A. skirrowii* sequence LRUX01000036.1. However, all genes mapped with a low sequence identity (49.9 to 87%). MLST results were extracted from the WGS data and were assigned to new sequence types (ST) (Supplementary Table 3), which were published within the PubMLST.org (ID 888-892). Verification of the results by traditional sequencing methods for MLST was not done.

The analysis of subsystem category distribution showed that the carbohydrate metabolism genes were comparable between *A. skirrowii* and *A. butzleri*. For *A. skirrowii* less elements could be classified for phages, prophages, transposable elements, plasmids, and a reduced number of genes related to potassium metabolism, iron metabolism, and iron acquisition.

## Discussion

The relevance of *Arcobacter* as a pathogen for humans has not yet been clarified, although three species including *A. butzleri*, *A. cryaerophilus*, and *A. skirrowii* have been associated with gastrointestinal diseases (Collado and Figueras, 2011). It can be assumed that the importance of *Arcobacter* in human infections is underestimated. For the isolation of *A. skirrowii* special cultivation procedures and therefore adequate detection and identification methods are not available in many laboratories. The taxonomic analysis of WGS data based on reads (MetaPhlAn and Kraken) also relies on yet incomplete databases, so most bioinformatics methods will not detect *A. skirrowii.* The taxonomic assignment based on assemblies and annotation with PhyloPhlAn proved as a fast and efficient method. PhyloPhlAn is a method using >400 proteins optimized from among 3,737 genomes thus reflecting more functional differences than phylogenies based on nucleotides.

Data on antimicrobial susceptibility of *A. butzleri* or *A. cryaerophilus* are scarce and almost lacking for *A. skirrowii* isolates. *A. skirrowii* seems to be susceptible to many antimicrobials (Houf et al., 2001). Although microdilution assays are usually favored, antibiotic testing is not standardized yet and the gradient strip method seems to be a more robust approach for these bacteria (Van den Abeele et al., 2014).

Antibiotic resistance can be induced by gene transfer or plasmid transfer events for example between human, animal, and plant-associated bacteria with streptomycin (Sundin and Bender, 1996). *A. butzleri* or *A. cryaerophilus* isolates from Europe (Van den Abeele et al., 2016) showed susceptibility to gentamicin, tetracycline, erythromycin, ciprofloxacin, and doxycycline and sometimes resistance to ampicillin. The here studied *A. skirrowii* isolates were more sensitive and showed only resistance to streptomycin. The CDC reported resistance to ciprofloxacin for almost 25% of human *Campylobacter* isolates and 2% were resistant to azithromycin (CDC, 2015). The antibiotic resistance of the *A. butzleri* isolate could not be predicted with the applied bioinformatics tools.

Antibiotic resistance is often mediated by plasmids especially in the *Enterobacteriaceae* family (Nikaido, 2009). Plasmids are commonly present in diverse prokaryotes, play an important role in the genetic evolution and adaptation of bacteria and were reported in 9.9% of *A. butzleri* isolates (Harrass et al., 1998; Toh et al., 2011). Plasmids could be neither predicted by the PlasmidFinder nor by visual inspection of the graphical assembly graphs (Wick et al., 2015) for any of the isolates. In agarose gels no plasmids could be detected comparable with Becker et al. (2016).

Multilocus Sequence Typing is a traditional technique that characterizes isolates using DNA sequences of multiple housekeeping genes. All isolates were assigned to distinct alleles and could be defined as new ST (Supplementary Table 3). Although no virulence factors could be predicted for these *Arcobacter* isolates, four genes known to contribute to host adherence and invasion could be mapped with low sequence identity (Levican et al., 2013). The low sequence identity was also an indication that *A. skirrowii* might show more variability within the nucleotide sequences which was not reflected in the databases, yet.

## Conclusion

In conclusion, *A. skirrowii* is a known, but rarely detected pathogen. The main reasons might be slow growth on culture media, overgrowth by other bacteria and underrepresentation in databases. Procedures in most routine microbiology laboratories need to be adapted for the detection and identification of this pathogen. Antibiotic susceptibility testing of *A. skirrowii* is preferably done using the gradient strip method due to the fastidious growth of the bacteria. Prediction of antibiotic susceptibility based on WGS data should be treated with caution.

Resistance to erythromycin, tetracycline, and streptomycin was found only in *A. butzleri*, while *A. skirrowii* was only resistant to streptomycin. Macrolides (here represented by erythromycin) are the preferred therapeutic agents in *Campylobacter* infections, but they are not necessarily first-choice antibiotics for *Arcobacter* infections for which tetracycline was proposed for severe cases only (Yan et al., 2000; Kayman et al., 2012; Arguello et al., 2015). An important factor in the development of resistance to antimicrobial agents is the uncontrolled use of antibiotics in animal husbandry. Monitoring and reporting of antimicrobial resistance data as well as WGS data analysis of *Campylobacter* and *Arcobacter* from domestic animals are important to monitor the evolution of antimicrobial resistance and to optimize diagnostics.

Here, solutions for diagnostic problems working with *A. skirrowii* were evaluated. Cultivation protocols were provided, MALDI-TOF MS spectra were made available and sequencing data were published in the NCBI, so that the software tools based on the RefSeq (such as BLAST and Kraken) will allow quick identification. The usage of open source software allows an economic and transparent application of the here established analysis. The investigated isolates were deposited in the open collection of the DSMZ. Our data can improve the diagnostic capabilities also of other laboratories and contribute to future work on epidemiological, pathogenetic, and functional analysis of these rarely recognized bacteria. This may further help to elucidate the mechanisms underlying the pathogenicity of *A. skirrowii.* The comprehensive bioinformatics analysis allows optimizing database dependent bioinformatics tools (for example the ResFinder, PubMLST, or Kraken). The analysis of the function of antibiotic resistance of new developing resistances is important to be able to compare the genetic make-up of resistant and susceptible strains. It can be speculated that the efforts undertaken to eradicate *Campylobacter* spp. (as cause of gastroenteritis) from the microbiome of farmed animals will lead to a replacement by *Arcobacter* spp. Then an assessment of the pathogenicity of *Arcobacter* spp. will be crucial.

## Author Contributions

IH, HH, AB, and HT have jointly conceived the study. IH provided strains, strain information, and metadata and antibiotic testing to the samples. AB performed bioinformatics analysis of genomes, assembly, and phylogenetic relationship.

## Conflict of Interest Statement

The authors declare that the research was conducted in the absence of any commercial or financial relationships that could be construed as a potential conflict of interest.

## Footnotes

1 https://www.dsmz.de
