# Supplementary material for: Antimicrobial Susceptibility and Genomic Structure of Arcobacter skirrowii Isolates
Source: Front Microbiol. 2018 Dec 14;9:3067. doi: 10.3389/fmicb.2018.03067 (PMC6302008; doi:10.3389/fmicb.2018.03067)
Supplement: Supplementary file 1 [file Data_Sheet_1.pdf]

Supplementary Material

Antimicrobial susceptibility and genomic structure of *Arcobacter skirrowii* isolates

Ingrid Hänel[1], Helmut Hotzel[1], Herbert Tomaso[1], Anne Busch[1]*

[1]Friedrich Loeffler Institut Jena, Institute of Bacterial Infections and Zoonoses (IBIZ), Germany

*** Correspondence:** Corresponding Author: anne.busch@fli.de

Frontiers in Microbiology, Brief Research Report, word count: 1999

Keywords: *Arcobacter skirrowii*, Arcobacter butzleri, Antimicrobial susceptibility, genomic structure

1 Supplementary Tables

Supplementary Table 1: Whole-genome sequences of 5 *A. skirrowii* and one *A. butzleri*, assembled and annotated

	17-1201-3 *A. skirrowii*	17-1201-4 *A. skirrowii*	17-1206-2 *A. skirrowii*	17-1208-1 *A. skirrowii*	17-1208-2 *A. skirrowii*	17-1168 *A. butzleri*
Mean coverage/standard deviation	29/20	216/151	191/130	176/118	187/129	190/89
GC-content [%]	27.7	27.6	27.6	27.6	27.6	26.9
Contigs	145	36	32	38	37	35
Bases	1911841	1938135	1937841	1940887	1938178	2177711
rRNA	3	3	3	3	2	2
repeat region	1	1	1	1	1	1
CDS	1937	1985	1986	1986	1972	2143
tRNA	42	42	42	42	42	46
tmRNA	1	1	1	1	1	1

Supplementary Table 2: Quality assessment with QUAST 4.3 [18] of the assemblies (SPAdes 3.9.1 (--careful) [17]) after filtering

Assembly	17-1168	17-1201-3	17-1201-4	17-1206-2	17-1208-1	17-1208-2
# contigs	36	172	78	61	75	1027
Largest contig	307398	86985	564516	564280	564280	547533
Total length	2182474	1930412	1962433	1953644	1962823	2530097
GC (%)	26.96	27.77	27.76	27.73	27.80	31.00
N50	179114	27335	131881	131881	131881	142808
N75	81358	13174	79986	79986	79986	15180
L50	5	23	4	4	4	4
L75	11	49	8	8	8	14
# N's per 100 kbp	0.00	0.00	0.00	0.00	0.00	0.00

Supplementary Table 3: All sequence types were new in Multilocus Sequence Typing (MLST) (Jolley and Maiden, 2010; Carattoli et al., 2014; Hunt et al., 2017). Several previously unknown loci are marked with an asterisk.

	ID, pubmlst.org	aspA	atpA	glnA	gltA	glyA	Pgm	tkt
17-1201-3 *A. skirrowii*	888	186*	103	95*	106*	286*	311*	135*
17-1201-4 *A. skirrowii*	889	186*	103	95*	106*	286*	311*	135*
17-1206-2 *A. skirrowii*	890	186*	103	93*	106*	286*	311*	135*
17-1208-1 *A. skirrowii*	891	186*	103	93*	106*	286*	311*	135*
17-1208-2 *A. skirrowii*	892	127	103	93*	106*	383*	311*	135*
17-1168 *A. butzleri*	893	69*	12	9	19	410	290	165

Supplementary Table 4: Exemplary MALDI-TOF MS masslist of a wildtyp *Arcobacter skirrowii* 1208_2

m/z	Intens.
2312.0	3622
2319.5	1383
2762.5	1697
2852.4	2242
3002.7	2482
3355.0	1257
3401.3	2382
3430.6	2723
3561.4	4325
3570.9	2149
3578.1	2567
3767.4	1715
4193.9	1702
4292.1	2046
4386.6	1072
4479.0	2674
4503.9	27851
4577.0	1791

4607.7	6609
4623.3	47505
4638.8	12730
4670.5	4252
4693.4	4636
5019.7	4397
5031.8	6226
5139.5	1364
5187.3	1394
5245.3	1620
5688.0	1416
5703.5	6418
5765.6	1212
6367.8	1326
6645.3	1544
6800.7	3442
6859.8	3713
7066.9	1006
7121.0	10999
7139.7	4350

7153.4	5619
7529.3	10479
7602.6	1046
7980.0	1422
7991.9	1420
8146.4	895
8173.5	2240
8385.3	2633
8582.4	2354
8639.3	1105
8772.0	1375
9006.5	38826
9151.7	846
9340.0	2968
9385.6	4925
9879.6	1304
10061.6	3944
10373.1	830
11529.0	620
15053.6	3473

15961.8	765
16343.1	842
