# Supplementary figures and images for: Antimicrobial Susceptibility and Genomic Structure of Arcobacter skirrowii Isolates
Source: Front Microbiol. 2018 Dec 14;9:3067. doi: 10.3389/fmicb.2018.03067 (PMC6302008; doi:10.3389/fmicb.2018.03067)

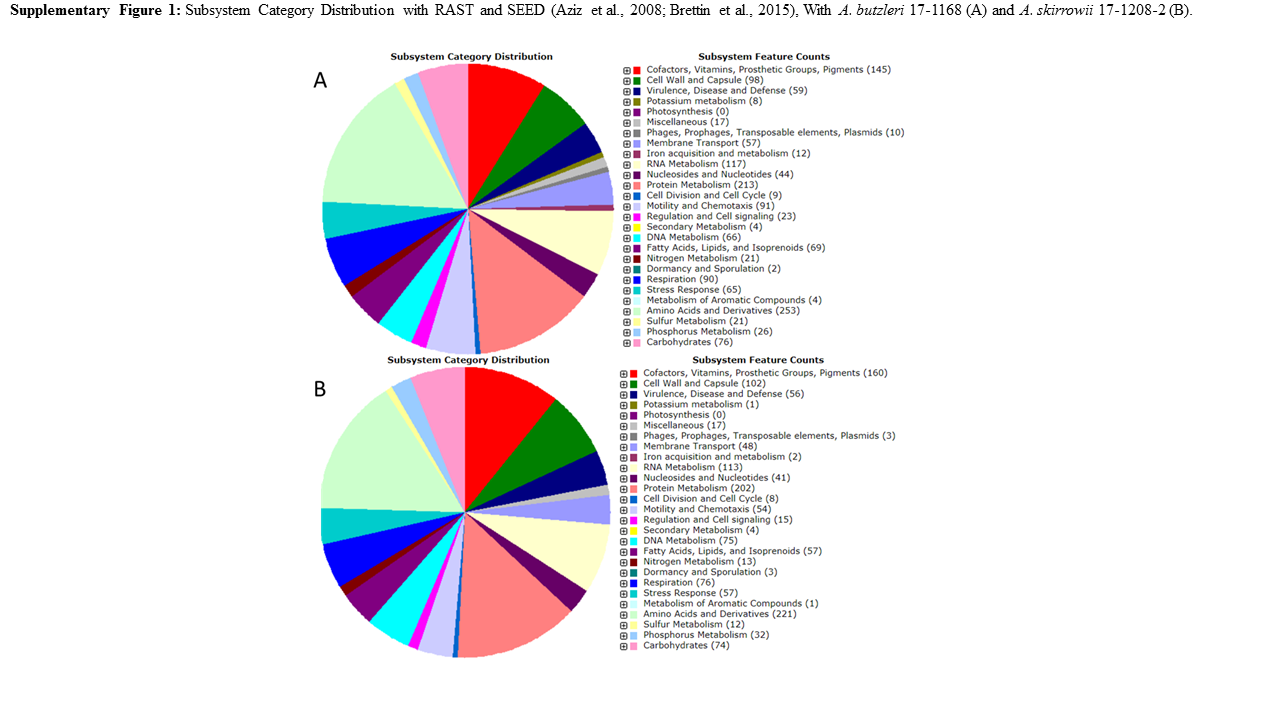

Supplement: Supplementary file 2 [file Image_1.TIF]
